# Fuzzy risk assessment of a deeply buried tunnel under incomplete information

**DOI:** 10.1098/rsos.180305

**Published:** 2018-10-03

**Authors:** Yuanpu Xia, Ziming Xiong, Hao Lu, Zhu Wen, Chao Ma

**Affiliations:** 1 State Key Laboratory of Disaster Prevention and Mitigation of Explosion and Impact, The Army Engineering University of PLA, Nanjing 210007, People's Republic of China; 2 School of Mechanical Engineering, Nanjing University of Science and Technology, Nanjing 210094, People's Republic of China

**Keywords:** fuzzy risk assessment, tunnel, similarity measure, information fusion, evidence theory

## Abstract

Risk assessment has always been an important part of safety risk research in tunnel and underground engineering. Owing to the characteristics of tunnel construction, to achieve an expected risk control effect, it is necessary to carry out accurate risk assessment research according to the risk assessment concept based on the entire tunnel construction process. At present, because of the frequent occurrences of safety accidents, a variety of risk assessment models have been proposed for different tunnel projects such as subways and railway tunnels, which can be roughly classified into two types: probability-based and fuzzy set theories. However, the existing models may be more suitable for the construction stage, and the design stage lacks a reliable and practical fuzzy risk assessment method. Therefore, based on fuzzy set theory and similarity measure theory, a risk assessment model is proposed to adapt to the characteristics that the risk information is difficult to quantify the fuzziness in the design phase. Firstly, new ideas of fuzzy risk analysis are proposed to overcome deficiencies in existing methods; secondly, a new similarity measure is constructed; then fusing multi-source fuzzy information based on evidence theory, the relationship between similarity measure and mass function is established. Finally, the new method is applied to the Yuelongmen tunnel. Results show that the concept of risk control and the risk assessment model are feasible.

## 1. Introduction

Owing to the characteristics and the potential application value of tunnel projects, tunnels are often required in the construction of infrastructure. For example, to build highways and high-speed railways, many deeply buried, long tunnels in southwestern China have been built or are under construction. A growing number of Chinese cities are building metro systems to solve their traffic congestion problems [1–3]. Tunnel construction involves the geotechnical engineering of large-scale complex systems, and various types of risk involved throughout the construction period [4,5]. This affects all parties involved as well as those not directly involved in the project [6]. Especially in geologically complex areas (karst developed areas, broken fault zones, areas of high crustal stress, etc.), various accidents such as collapses [7], water in-rushes [8] and rock bursts [9] can easily be triggered, which may cause casualties, economic loss, environmental harm and delay which may eventually lead to cost overruns and contract extensions [10]. The frequent occurrence of disasters in tunnel construction is increasingly arousing the attention of all parties including the public [11]. Therefore, how to reduce the occurrence of accidents during tunnel construction, decrease the number of casualties and mitigate future economic losses has always been a focus for engineers, especially in developing countries such as China.

Risk assessment is an important part of risk management, which is a tool designed to support all aspects of decision-making from the beginning to the end of a process [12]. It has been increasingly valued and regarded as an important guarantee for the safe running of large engineering projects [13]. Various risk assessment tools have been applied in tunnel engineering, including security vulnerability analysis (SVA) [14], bow-tie diagrams [15], event tree analysis (ETA) [16], fault tree analysis (FTA) [17], probabilistic risk analysis [18,19], the analytical hierarchy process (AHP) [20], grey systems [21], Bayesian networks [22] and fuzzy sets [23]. In addition, artificially intelligent methods such as geographic information systems (GIS) [24], risk analysis software [25,26] and Monte Carlo simulations [27] have also been widely used in recent years. Probabilistic risk assessment (PRA) is the method most widely used in geotechnical engineering [13]. For example, Jurado *et al*. [28] developed a general PRA framework to estimate the probability of occurrence of groundwater-related hazards in tunnel construction based on FTA. Sousa & Einstein [29] introduced a dynamic Bayesian network (DBN) model for the risk of construction failure. Šejnoha *et al*. [30] estimated the probability of the occurrence of cave-in collapses and their consequences using FTA and ETA tools. Špačková *et al*. [31,32] further put forward a probabilistic model by considering the inhomogeneity of the geological conditions, the uncertainty of the consequences of damage, and human and other external factors.

With the increasing application of risk assessment models in tunnel engineering, some scholars gradually realized that insufficient information is a major problem facing risk analysts working in geotechnical engineering contexts, and the usual methods cannot support precise probabilities [33]: for example, Marques *et al*. [34] proposed that the conventional probabilistic approach to uncertainty can be extended to include imprecise information in the form of intervals. Beer *et al*. [35] have realized the role of epistemic uncertainty in civil engineering reliability evaluation and discuss the relationship between subjective probability and imprecise probability that represents epistemic uncertainty. To deal with imprecise information (insufficient information, vague information, descriptive language information, incomplete data, etc.), some fuzzy risk assessment models based on fuzzy set theory (first proposed by Zadeh [36]) have been proposed. The fuzzy risk assessment models can be roughly divided into the following categories:

(1) Fuzzy risk assessment function analysis model. The mechanism underpinning the development and occurrence of a geological hazard is analysed using a physico-mechanical model, then the risk assessment function is constructed, and the value of the function parameters is fuzzed. For example, firstly, the mechanical model of a breakwater failure problem is established, then extended fuzzy set theory (possibility theory and evidence theory) is used to evaluate the risk to the breakwater [37]. Hao *et al*. [38] first analysed the mechanism of water in-rush in karst tunnels, and then used margin theory to determine the risk of water in-rush under epistemic uncertainty.
(2) Mixed risk assessment model. The existing risk assessment methods such as Bayesian network, or FTA, are fuzzed using fuzzy set theory, and a fuzzy risk assessment model is obtained. For example, Zhang *et al*. [39] proposed a fuzzy probability risk decision approach for metro construction based on fuzzy set theory and FTA. Nadjafi *et al*. [40] proposed a dynamic FTA based on a fuzzy lower and upper (L-U) bounded failure distribution. To deal with the uncertainty during the assessment of water in-rush risk in underground engineering works, Yang *et al*. [41] constructed a model based on GIS and the fuzzy set theory. In addition, a Bayesian network is seen as a common tool used to deal with risk analysis, and different fuzzy Bayesian network assessment and decision models are often suggested as being applicable [42–45].
(3) Risk assessment model based on attribute measure. Wang *et al*. [46] and Chu *et al*. [47] constructed a risk assessment model of water in-rush in a karst tunnel and a coal mine based on the secondary fuzzy comprehensive evaluation, respectively, where every index was constructed by membership degree. Li *et al*. [48] proposed a risk assessment model of water in-rush in a karst tunnel based on attribute measure theory.
(4) Fuzzy risk assessment model based on a similarity measure. This was first introduced by Wang in 1983 [49] and has been applied in many fields. Similar measures indicate the degree of similarity between two fuzzy numbers. Yong *et al*. [50] proposed a similarity measure-based method to estimate human-health risk from groundwater contamination and evaluated possible regulatory actions. Parandin *et al*. [51] proposed a fuzzy risk analysis algorithm to cope with fuzzy analysis problems based on a new similarity measure.

In addition, the situation where random and fuzzy variables exist in the same risk assessment model may occur causing hybrid uncertain information. For example, Xie *et al*. [52] developed a new hybrid reliability analysis method so that the probability analysis (PA) and interval analysis (IA) loops are decomposed into two separate loops. The existence of hybrid uncertain information increases the complexity of the risk assessment model, and so Peng *et al*. [53] proposed a hybrid first order reliability analysis method to improve reliability analysis of the results.

From this summary, we can see that risk assessment has aroused widespread interest among geotechnical engineers. Different risk assessment models are regularly proposed, and fuzzy risk analysis is attracting more attention, but we find that the current research into risk in tunnel engineering still has the following deficiencies.

### 1.1. The study of risk assessment lacks communication with decision-makers

Risk management is essentially a series of risk decision-making processes, and risk assessment should also consider how to be more conducive to decision-making [12]. Risk analysts and decision-makers generally belong to different teams, which complicates the work related to risk control [54]. For complex systems engineering such as the construction of mountain tunnels, the security issues are generally more complex, in addition to considering the severity of the risk, the decision process often needs to consider the impact of other factors. For example, the risk attitude, work experience and educational background of decision-makers may affect the level of decision-making. In addition, decision-makers always want to achieve the best risk control effect under the constraints of limited cost, time and human resources. Therefore, to better facilitate subsequent decision-making, it is necessary for risk analysts to consider the influence of decision factors when conducting risk assessments.

### 1.2. The feasibility of the risk assessment model is not fully considered

During construction, risk assessment work is implemented dynamically as the construction processes: in terms of time, economy, human resources and so on, will be subject to certain restrictions. No project will invest unlimited resources in risk assessment work. Furthermore, a mountain tunnel will be affected by the construction conditions, natural environmental changes such as earthquakes, rainstorms, etc. The literature [42] emphasizes the role of an expert group in the use of fuzzy Bayesian networks; due to the advantages of using experts, they are helpful when improving the reliability of risk assessment. However, it is unrealistic to get expert help and guidance at every turn: it is not always necessary to invite expert opinion.

### 1.3. The study of risk assessment lacks continuity

Current models are mainly aimed at the safety and risk during construction, but the construction of a tunnel involves investigation, design and construction stages. Although the risk characteristics of different stages are different, they remain closely linked. Risk assessment during the construction period is not independent of that at other stages and should be carried out on the basis of risk assessment in the design stage. Therefore, we should consider how to carry out risk assessment over the whole construction cycle.

**Figure 1.**
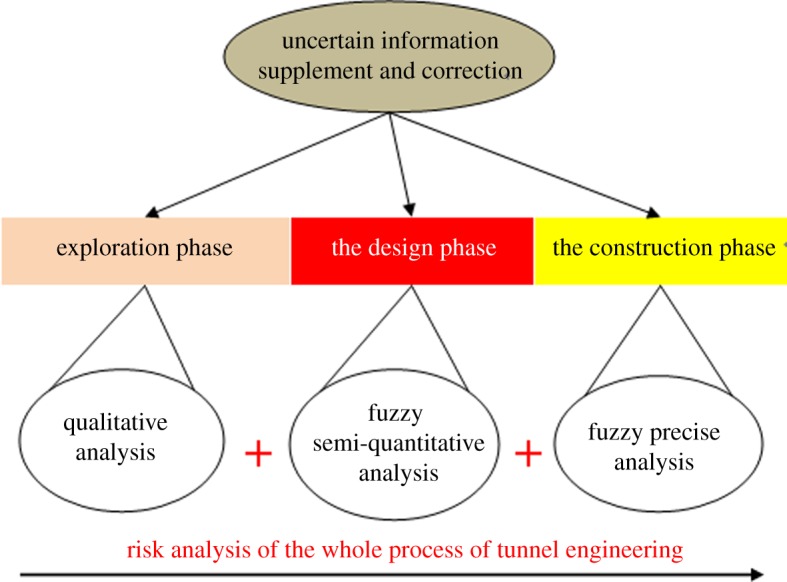
Risk assessment system for use in tunnel engineering.

Based on the above analysis, we believe that it is necessary to carry out the risk assessment over the entire process of tunnel construction. It can adapt to the characteristics of tunnel construction and also fully use the impact of information supplement on risk assessment to improve the science and rationality of risk assessment results. Through the risk analysis of the whole construction process, we can get to know the tunnel risks and focus on the key goals. This process can reduce the error of decision-making, improve the utilization of resources and increase the efficiency of decision-making. We constructed a risk analysis model aiming at the whole construction process of tunnel engineering, as shown in figure 1.

From figure 1, the exploration phase is the initial evaluation, and it mainly entails qualitative analysis. It is important to initially grasp the engineering geological and hydrogeological conditions in the area where the tunnel may pass, the route selection and its feasibility, avoidance of geologically complex areas, such as faults, karst, underground rivers, etc. In the design stage, based on the site investigation, the geological conditions are analysed further, the risk of the major geologic hazards and their distribution along the route are judged, and corresponding risk control plans are formulated. Owing to the characteristics of deep tunnels, the geological information in this stage is not sufficient. Fuzzy semi-quantitative analysis may be better, although semi-quantitative evaluation cannot give accurate results. The risk assessment results can be divided into several levels (typically four or five), the entire line of tunnel can be divided into different risk grade sections according to the above risk grades, and then risk control plans are formulated which are feasible in cost and time. There are more risk factors and more sources of uncertainty in risk assessment during construction. If the quality of risk assessment result is not high enough, accidents may happen. However, the construction stage is constrained by various factors, and the resources available are also limited, which means that we have to put limited resources into the most in-need sections, namely high-risk areas. Then, the precise evaluation models described above can be used to evaluate these high-risk sections. Through continuous and dynamic risk assessment research, we can realize the target risk levels at limited economic, time and human resource cost. Risk can be reduced to an acceptable level. Of course, the realization of this goal is inseparable from the reliable and practical risk assessment results of the design stage.

At present, various risk analysis methods based on fuzzy set theory have been proposed. Different methods have their characteristics and limitations, e.g. the fuzzy risk assessment function analysis model must construct a specific function; mixed risk assessment involves the integration of different expert opinions; the risk assessment levels have been fuzzed in attribute measure-based risk analysis models, but the risk index is regarded as a precise value; the study of similarity measure-based fuzzy risk analysis has aroused wide interest in different fields and has evolved continuously [55]. By combining with the characteristics of risk assessment at the design stage, evaluation results should be reliable and easy to understand for decision-makers, and the calculations in the model should not be too complicated. Considering the above factors, we believe that the fuzzy risk assessment model, based on a similarity measure, is suitable for risk analysis at the tunnel engineering design stage.

This paper is organized as follows: an improved research idea of risk assessment has been proposed based on the research status of similar measure-based fuzzy risk analysis in §2. In §3, based on the deficiency of existing calculation methods of similarity measure, an improved formula is proposed. In §4, firstly, the similarity measure is transformed into a form of evidence (i.e. a mass function), and then based on the theory of evidence synthesis, different pieces of evidence are synthesized to obtain the assessment result. In §5, the proposed risk assessment model is applied to Yuelongmen Tunnel. The results show that the proposed method is effective and can be used as an important part of the tunnel safety risk assessment system. The main conclusions are drawn in §6.

## 2. Risk assessment model

### 2.1. The traditional fuzzy risk assessment method based on similarity measure

Although there are differences between existing fuzzy risk analysis models, the basic processes therein are similar, and the typical steps are shown below:

*Step 1*. Construction of a set of linguistic terms and its corresponding generalized trapezoidal fuzzy numbers. For example, the risk is divided into nine levels (table 1).
*Step 2.* Description of the likelihood and severity of each evaluation index with linguistic terms.
*Step 3.* Computation of the total risk *R* of event A with the help of arithmetic operators used on generalized trapezoidal fuzzy numbers.
  2.1 $$\tilde{R}=\frac{\sum_{i=1}^{n}{\tilde{w}}_{i}⊗{\tilde{R}}_{i}}{\sum_{i=1}^{n}{\tilde{w}}_{i}},$$
where *R̃* is described by a generalized trapezoidal fuzzy number.
*Step 4.* Measuring the similarities of total risk *R̃* with all given linguistic terms in Step 1, and considering the largest similarity as a risk value of the system in linguistic terms.

**Table 1.** Fuzzy representations of linguistic terms [56].

	linguistic valued trapezoidal fuzzy number		linguistic valued trapezoidal fuzzy number
absolutely low	(0, 0, 0, 0, 1.0)	fairly high	(0.58, 0.63, 0.80, 0.86, 1.0)
very low	(0, 0, 0.02, 0.07, 1.0)	high	(0.72, 0.78, 0.92, 0.97, 1.0)
low	(0.04, 0.1, 0.18, 0.23, 1.0)	very high	(0.93, 0.98, 1.0, 1.0, 1.0)
fairly low	(0.17, 0.22, 0.36, 0.42, 1.0)	absolutely high	(1.0, 1.0, 1.0, 1.0, 1.0)
medium	(0.32, 0.41, 0.58, 0.65, 1.0)		

From the above steps, we can see that the model involves operations between different fuzzy numbers (see appendix A for details) and the acquisition of a similarity measure. However, the results of the multiplication and division with the existing formulae are an approximation of a triangular fuzzy number for simplified computation consideration, which is likely to result in partial information loss of the obtained risk value [57]. In the case of more risk indicators, it may have a greater impact on the results of risk assessment. Additionally, although different formulae are used to obtain the similarity measure, the calculation between nonlinear fuzzy numbers is still not ideal. For the fuzzy risk analysis of a tunnel, the fuzzy numbers of nonlinear parameters may be more reasonable. Therefore, to improve the reliability of tunnel engineering risk assessment, it is necessary to improve the existing risk analysis model.

### 2.2. The improved risk assessment model

To solve the above problems, we can improve the calculation formula of similar measures. Then to avoid the problem of missing information, we hope that the constructed model does not involve the computation of fuzzy numbers. The risk assessment process involves the processing of information about different risk factors and generation of a reasonable, comprehensive, evaluation based on all available information. To some extent, the process of risk assessment can be regarded as a process of multi-source information fusion.

Evidence theory [58] is often used in information fusion applications. An important part of evidence theory is how to construct the mass function, which represents the degree of evidential support for an event. In fuzzy risk analysis, it is the degree of support of the risk factor information to the risk event belonging to each risk grade. The similarity measure indicates the degree of similarity between two fuzzy numbers, if the risk factor information is expressed as a fuzzy number (i.e. a membership function) and the risk levels can be expressed by other fuzzy numbers, then the similarity measure can be considered as the degree of support given by the risk factor information to the risk level. Therefore, there is some connection between the similarity measure and the mass function, and it is reasonable to deduce the mass function of evidence theory by a similarity measure. Therefore, we can first calculate the similarity measure between the risk assessment index and different risk levels; then the assessment result can be determined by evidence theory to fuse multi-source information. The specific steps required are as follows.

*Step 1*. Construction of a set of linguistic terms and its corresponding generalized trapezoidal fuzzy numbers. For risk analysis in tunnel engineering, this is generally divided into four risk levels, which is different from the nine levels described above. Referring to the classification of the existing tunnel water in-rush risk assessment level [59], the risk levels are as listed in table 2.

**Table 2.** Risk levels for water in-rush risk evaluation in a tunnel.

risk level	linguistic valued trapezoidal fuzzy number
A	(0, 0, 15, 21; 1; 1, 0.5)
B	(15, 21, 37, 45; 1; 0.5, 2)
C	(37, 45, 58, 71; 1; 0.5, 1)
D	(58, 71, 100, 100; 1; 1, 1)

Table 2 shows a percentage system, which is mainly used for convenience. The risk level can be represented by membership functions. The equation describing level A is written as follows:

2.2 $$u_{A}(x)=\{\begin{array}{ll} 1, & 0\leq x\leq 15\\ {\left(\frac{21-x}{6}\right)}^{{\;}^{0.5}}, & 15\leq x\leq 21\\ 0, & \mathrm{others} \end{array}.$$

*Step 2.* Determination of the fuzzy number of each index.

For the fuzzy risk analysis based on generalized trapezoidal fuzzy numbers, the fuzzy numbers are mainly chosen from table 1 according to different linguistic terms; however, here we do not require fixed fuzzy numbers; we construct different fuzzy numbers according to different research objects and risk information. The risk assessment of water in-rush in tunnel engineering is taken as an example to discuss how to deal with fuzzy information about risk factors. The specific construction process for incomplete data is presented as follows:

(1) Assuming that *X* = {*x*_1_, *x*_2_, … , *x_n_*} is a set of data describing the uncertainty of a risk factor, the average value is:
  2.3 $$m=\frac{1}{n}\sum_{i=1}^{n}x_{i}.$$
(2) The data are re-divided by the mean, and two datasets are obtained:
  2.4 $$X_{1}=\{x_{1},x_{2},\ldots ,x_{n}|x_{i}<m\},\;X_{2}=\{x_{1},x_{2},\ldots ,x_{n}|x_{i}>m\}.$$
The average values of the new two datasets are then presented as:
  2.5 $$m_{1}=\frac{1}{N(X_{1})}\sum_{i=1}^{n}x_{i},x_{i}<m,\;m_{2}=\frac{1}{N(X_{2})}\sum_{i=1}^{n}x_{i},x_{i}>m.$$
(3) The membership function of the fuzzy number is:
  2.6 $$u(x_{i})=\{\begin{array}{ll} \left[\frac{x_{i}-(2m_{1}-m+1)}{2(m-m_{1}-1)}\right]m, & 2m_{1}-m+1\leq x_{i}<m-1\\ 1, & m-1\leq x_{i}<m+1\\ {\left[\frac{(2m_{2}-m-1)-x_{i}}{2(m_{2}-m-1)}\right]}^{2}, & m+1\leq x_{i}<2m_{2}-m-1\\ 0, & \mathrm{others} \end{array}.$$

The values of *m* and *n* can be determined according to the actual situation of the risk indicator information. Thus, the constructed possibility distribution can objectively reflect uncertain information.

*Step 3.* The determination of similarity measure between risk indicator information and different risk levels. The specific formula used in this calculation is discussed in §3.

*Step 4*. Deriving the initial mass function from the similarity measure.

*Step 5*. Adjustment of initial mass function.

*Step 6*. Multi-source uncertainty information fusion. The evidence fusion formula is:

2.7 m(A)={0,A=Φ11−k∑Ai,BjAi∩Bj=Am1(Ai)m2(Bj),A≠Φ.

where *A*, *A_i_*, *B_j_* ∈ 2*^θ^* and $k=\sum_{A_{i}\cap B_{j}=\Phi }m_{1}(A_{i})m_{2}(B_{j})$: Steps 4 and 5 will be elaborated in §4.

## 3. Calculation of similarity measure

### 3.1. Existing similarity measures and their limitations

The similarity measure between fuzzy numbers is an important research topic in the fuzzy risk analysis, and it has aroused the interest of many scholars. Different similarity measures have been proposed by Chen & Chen [60,61], Wei & Chen [62], Xu *et al.* [63] and Hejazi *et al*. [64] (see appendix B for details). Then, in 2015, Patra & Mondal [56] proposed a new similarity measure associating the geometric distance, area and height of generalized trapezoidal fuzzy numbers (equation (3.1)), which overcomes the shortcomings of previous similarity measures:

3.1 $$S(\tilde{A},\tilde{B})=\left(1-\frac{\sum_{i=1}^{4}\left|a_{i}-b_{i}\right|}{4}\right)\times \left(1-\frac{1}{2}\{\left|A(\tilde{A})-A(\tilde{B})|+|w_{\tilde{A}}-w_{\tilde{B}}\right|\}\right).$$

Although the above equation is improved on previous research, this method still has two significant defects; therefore, Khorshidi & Nikfalazar [65] proposed the following improved calculation method:

3.2 $$\begin{array}{l} S(\tilde{A},\;\tilde{B})=\left(1-\frac{\sum_{i=1}^{4}\left|a_{i}-b_{i}\right|}{4}\times d^{\prime }(\tilde{A},\;\tilde{B})\right)\;\\ \;\times \left(1-\frac{\left|A(\tilde{A})-A(\tilde{B})|+|w_{\tilde{A}}-w_{\tilde{B}}|+(|P(\tilde{A})-P(\tilde{B})|)\text{/​}\max(P(\tilde{A}),\;P(\tilde{B})\right)}{3}\right). \end{array}$$

If $\max(P(\tilde{A}),P(\tilde{B}))=0$, then we have $\left(P(\tilde{A})-P(\tilde{B})\right)/\max(P(\tilde{A}),P(\tilde{B}))=0$.

In addition, Li & Zeng [66] proposed a new similarity measure between generalized trapezoidal fuzzy numbers, which could reduce the complexity of computation and improve their ability to distinguish between different values, thus broadening its scope of application:

3.3 $$S(\tilde{A},\tilde{B})=se\times sp,$$

where

$$se=\{\begin{array}{ll} e^{-|a_{1}-b_{1}|}, & a_{1}=a_{4},\;b_{1}=b_{4}\\ e^{-(k+h+s+z)}, & \mathrm{otherwise} \end{array}\;\mathrm{and}\;sp=\frac{\varepsilon +\min(P(\tilde{A}),P(\tilde{B}))}{\varepsilon +\max(P(\tilde{A}),P(\tilde{B}))},$$

*k* is the span difference, *h* is the centre width difference, *s* represents the minimum value of the difference between the left- and right-hand points of the peak value and *z* is the central difference for the fuzzy numbers *Ã* and *B̃*, respectively. The specific formulae are as follows:

$$\begin{array}{rl} & k=|\left(a_{4}-a_{1})-(b_{4}-b_{1}\right)|,h=|\left(a_{3}-a_{2})-(b_{3}-b_{2}\right)|,\\ & s=\min(|a_{2}-b_{2}|,|a_{3}-b_{3}|),z=\left|\frac{a_{4}+a_{1}}{2}-\frac{b_{4}+b_{1}}{2}\right|. \end{array}$$

**Figure 2.**
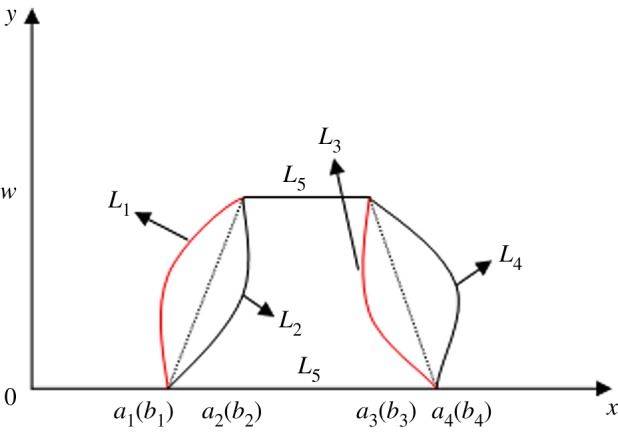
Membership functions of fuzzy numbers A and B.

Through the use of the above analysis, the related calculation method has been improved; however, the above methods are mainly used for generalized trapezoidal fuzzy numbers and cannot be used directly for nonlinear fuzzy numbers. For example, assuming the fuzzy numbers *Ã* and *B̃* are as shown in figure 2.

Owing to the nonlinear effect, although *a*_1_ = *b*_1_, *a*_2_ = *b*_2_, *a*_3_ = *b*_3_ and *a*_4_ = *b*_4_, fuzzy numbers *Ã* and *B̃* are different. In addition, the areas $A(\tilde{A})=A(\tilde{B})$, perimeters $P(\tilde{A})=L_{1}+L_{5}+L_{3}+L_{6},P(\tilde{B})=L_{2}\;+L_{5}+L_{4}+L_{6}$ and heights $w(\tilde{A})=w(\tilde{B})$. If the latest research results are taken to calculate the similar measures, namely equation (3.2) or (3.3), we find that the similarity measure is 1, which is inconsistent with reality. Therefore, it is necessary to propose another nonlinear similarity measure.

### 3.2. Proposed similarity measure for nonlinear fuzzy numbers

The calculation of the similarity measure generally considers the geometric distance, distance from the centre of gravity, perimeter, area, height and so on. Therefore, based on previous studies, especially on the latest research results, we propose the following similarity measure calculation method: suppose that there are two fuzzy numbers *Ã* and *B̃*, and the corresponding membership functions are $u(\tilde{A})$ and $u(\tilde{B})$, respectively, then:

3.4 $$S(\tilde{A},\tilde{B})=\left(1-\frac{\sum_{i=1}^{4}\left|a_{i}-b_{i}\right|}{4}\right)\times e^{-d^{\prime }(\tilde{A},\tilde{B})}\times \frac{\min(P(\tilde{A}),P(\tilde{B}))+\min(A(\tilde{A}),A(\tilde{B}))+\min(w_{\tilde{A}},w_{\tilde{B}})}{\max(P(\tilde{A}),P(\tilde{B}))+\max(A(\tilde{A}),A(\tilde{B}))+\max(w_{\tilde{A}},w_{\tilde{B}})}.$$

where $d^{\prime }(\tilde{A},\tilde{B})$ denotes the distance from the centre of gravity, $d^{\prime }(\tilde{A},\tilde{B})=\sqrt{{\left(x_{{\;}_{\tilde{A}}}^{*}-x_{{\;}_{\tilde{B}}}^{*}\right)}^{2}+{\left(y_{\tilde{A}}^{*}-y_{\tilde{B}}^{*}\right)}^{2}}$, $x_{\tilde{A}}^{*}$ is

3.5 $$x_{\tilde{A}}^{*}=\frac{\int_{a_{1}}^{a_{4}}xu_{\tilde{A}}dx}{\int_{a_{}}^{a_{4}}u_{\tilde{A}}d_{x}}.$$

Similarly, we can also calculate other coordinates $y_{\tilde{A}}^{*}$, $x_{\tilde{B}}^{*}$, $y_{\tilde{B}}^{*}$ of the centre of gravity. The perimeters and areas can be calculated thus:

3.6 $$P(\tilde{A})=(a_{4}-a_{1})+\int_{a_{1}}^{a_{4}}{\left(1+u_{\tilde{A}}^{2}\right)}^{1/2}dx$$

and

3.7 $$A(\tilde{A})=\int_{a_{1}}^{a_{4}}u_{\tilde{A}}\;dx.$$

A larger $S(\tilde{A},\tilde{B})$ corresponds to a closer similarity between fuzzy numbers *Ã* and *B̃*. It will be proved that the proposed similarity measure could satisfy some of the properties.

From equation (3.4), $S(\tilde{A},\tilde{B})\in [0,1]$ and $S(\tilde{A},\tilde{B})=S(\tilde{B},\tilde{A})$ are satisfied. Another property of the above formula is discussed. $\tilde{A}=\tilde{B}$ if and only if $S(\tilde{A},\tilde{B})=1$.

*Proof*. When $\tilde{A}=\tilde{B}$, it implies that $d^{\prime }(\tilde{A},\tilde{B})=0$, $P(\tilde{A})=P(\tilde{B})$, $A(\tilde{A})=A(\tilde{B})$, $w(\tilde{A})=w(\tilde{B})$, then we have $S(\tilde{A},\tilde{B})=1$.

If $S(\tilde{A},\tilde{B})=1$, it implies that $\sum_{i=1}^{4}|a_{i}-b_{i}|=0$, which means that *a*_1_ = *b*_1_, *a*_2_ = *b*_2_, *a*_3_ = *b*_3_, *a*_4_ = *b*_4_. $d^{\prime }(\tilde{A},\tilde{B})=0$, which means that $x_{\tilde{A}}^{*}=x_{\tilde{B}}^{*}$, and $y_{\tilde{A}}^{*}=y_{\tilde{B}}^{*}$. $\min(P(\tilde{A}),P(\tilde{B}))+\min(A(\tilde{A}),A(\tilde{B}))+\min(w_{\tilde{A}},w_{\tilde{B}})/\max(P(\tilde{A}),$ $P(\tilde{B}))+\max(A(\tilde{B}),A(\tilde{B}))+\max(w_{\tilde{A}},w_{\tilde{B}})=1$, which suggests that $P(\tilde{A})=P(\tilde{B})$, $A(\tilde{A})=A(\tilde{B})$ and $w(\tilde{A})=w(\tilde{B})$, Therefore, the fuzzy number *Ã* would be equal to the fuzzy number *B̃*.

## 4. Information fusion and risk evaluation

### 4.1. Construction of initial mass function

The mass function obtained by the similarity measure is the key step in multi-source information fusion. The main steps for deriving the mass function by similarity measure are shown below.

(1) Determination of mean *E_i_* and the variance *D_i_* for each type of uncertainty information.

4.1 $$E_{i}=M(A)=\int_{0}^{1}[\alpha_{1}(\lambda )+\alpha_{2}(\lambda )]\lambda \;d\lambda ,$$

where [*α*_1_(*λ*), *α*_2_(*λ*)] is the cut set of *λ* for the distribution of the fuzzy number.

4.2 $$D_{i}=\delta (A)=\int_{0}^{1}\frac{1}{6}{\left[\alpha_{2}(\lambda )-\alpha_{1}(\lambda )\right]}^{2}\lambda \;d\lambda .$$

Then the induced function *Q_i_* is obtained

4.3 $$Q_{i}=\frac{E_{i}}{D_{i}}.$$

(2) The ordered function Order*_i_* can be obtained by use of

4.4 $$\mathrm{Orde}r_{i}=\frac{Q_{i}}{\sum_{i=1}^{N}Q_{i}}.$$

(3) The degree of support for uncertainty information at each risk level can be determined based on

4.5 $$\mathrm{Sup}(j)=\mathrm{Orde}r_{i}\times S_{ij}\;,j=1,2,\ldots ,M,$$

where *S_ij_* represents the similarity measure between the *i*th risk factor information and the *j*th risk level.

(4) The credibility of uncertainty information to risk level can be obtained from the use of

4.6 $$Crd(j)=\frac{\mathrm{Sup}(j)}{\sum_{\;j=1}^{M}\mathrm{Sup}(j)},$$

where $\sum_{\;j=1}^{M}Crd(j)=1$, and *Crd*(*j*) can be seen as the basic trust assignment function *m*(*j*) (mass function) of uncertainty information assigned to a given risk level.

### 4.2. Adjustment of initial mass function

Different types of uncertain information can affect assessment results from different perspectives, but the degree of impact is different. In other words, the importance is different. The above-mentioned differences cannot be reflected in the initial mass functions. Therefore, it is necessary to modify the initial mass function. This section focuses on determining the weight of each indicator and how to use weights to influence mass functions.

#### 4.2.1. Determination of weights

To improve the reliability of each assigned weight, the influences of subjective and objective weights are considered simultaneously.

##### 4.2.1.1. Subjective weight

AHP is the main method for subjective weighting [67]; however, this method does not consider the correlation effect among different indices. For example, surface water catchment conditions can affect the water pressure at a tunnel face, thus, the trapezoidal fuzzy number is introduced to characterize the expert information. Suppose that the influence matrix of the risk factors is as shown in table 3.

**Table 3.** Influence matrix of the risk assessment indices.

	*u*_1_	…	*u_i_*	…	*u_n_*
*u*_1_	*m*_11_	…	*m*_1*i*_	…	*m*_1*n*_
⋮	· · ·	…	· · ·	…	…
*u_i_*	*m_i_*_1_	…	*m_ii_*	…	*m_in_*
⋮	· · ·	…	· · ·	…	…
*u_n_*	*m_n_*_1_	…	*m_ni_*	…	*m_nn_*

*m_ij_*(*i*, *j* ∈ (1, 2, 3, 4)) represents the degree of influence of index *u_i_* to index *u_j_* (the scale method and its definition of *m_ij_* are summarized in appendix C): generally, *m_ij_* ≠ *m_ji_*. Suppose that the trapezoidal fuzzy numbers given by the experts are *m_ij_* = (*l_ij_*, *e_ij_*, *f_ij_*, *s_ij_*), where *l_ij_* ≤ *e_ij_* ≤ *f_ij_* ≤ *s_ij_*. The expected value of the trapezoidal fuzzy number is:

4.7 $$E(a_{ij})=bE^{L}(m_{ij})+(1-b)E^{U}(m_{ij}).$$

$E^{L}(m_{ij})=(l_{ij}+e_{ij})/2$ and $E^{U}(m_{ij})=(f_{ij}+s_{ij})/2$ are the left and right expected values of the trapezoidal fuzzy numbers, respectively; *b* represents the attitude of the experts on the results (here, *b* = 0.5). The fuzzy influence value of risk index *u_i_* can be obtained using

4.8 $$u_{i}=\left(\frac{\sum_{i=1}^{m}l_{ij}}{\sum_{i=1}^{n}\sum_{\;j=1}^{n}s_{ij}},\frac{\sum_{i=1}^{n}e_{ij}}{\sum_{i=1}^{n}\sum_{\;j=1}^{n}\;f_{ij}},\frac{\sum_{i=1}^{n}\;f_{ij}}{\sum_{i=1}^{n}\sum_{\;j=1}^{n}e_{ij}},\frac{\sum_{i=1}^{n}s_{ij}}{\sum_{i=1}^{n}\sum_{\;j=1}^{n}l_{ij}}\right).$$

Then the subjective weight can be obtained:

4.9 $$w_{u_{i}}^{\overset{`}{\;}}=\frac{E(u_{i})}{\sum_{i=1}^{n}E(u_{i})}.$$

##### 4.2.1.2. Objective weight

The calculation process of the objective weight is presented below [68].

(1) The correlation coefficient matrix can be established as follows:
  $$R=\left[\begin{array}{llll} r_{11} & r_{12} & \ldots & r_{1n}\\ \vdots & \vdots & r_{ij} & \vdots \\ r_{n1} & r_{n2} & \cdots & r_{nn} \end{array}\right],$$

where *r_ij_*(*i*, *j* = 1, 2 … *n*) is the correlation coefficient of *u_i_*; *u_j_* and *r_ij_* can be represented by the following formula:

4.10 $$|r_{ij}|=\left|\frac{\mathrm{cov}(u_{i}(x),u_{j}(x))}{\sqrt{D(u_{i}(x))}\sqrt{D(u_{j}(x))}}\right|.$$

(2) The mean value of the correlation degree of *u_i_* is as follows:
  4.11 $${\bar{m}}_{i}=\frac{\sum_{\;j=1,j\neq i}^{n}\left|r_{ij}\right|}{\left(n-1\right)}.$$
(3) The greater the correlation of an index, the smaller its importance. Then the objective weight can be determined as follows:
  4.12 $$w_{u_{k}}^{**}=\frac{\psi_{k}}{\sum_{k=1}^{n}\psi_{k}},\;\psi_{k}=\frac{1}{{\bar{m}}_{k}}.$$

Then the comprehensive weight is

4.13 $$w_{i}=\alpha w_{i}^{*}+(1-\alpha )w_{i}^{**}$$

where *α* is the scale factor of the subjective and objective weights (here, *α* = 0.5).

#### 4.2.2. The improved mass function

When the weights are determined, the factor with the maximum weight is regarded as the key factor, while the others are regarded as non-key factors. The relative weights *β_i_* of each factor relative to the key factor are determined. The initial mass function is modified by using the relative weights. The specific process is detailed below:

(1) Suppose that $\alpha (x_{i})=\max w_{i}$, *x_i_* is the key factor and the relative weights *β_i_* can be obtained by using
  4.14 $$\beta_{i}=\frac{\alpha (m_{i})}{\alpha (m_{f})}.$$
(2) The modified equation of mass function is:
  4.15 $$\left\{ \begin{array}{l} {m_{i}}^{\overset{`}{\;}}(A)=\beta_{i}m_{i}(A)\\ {m_{i}}^{\overset{`}{\;}}(\Theta )=\beta_{i}m_{i}(\Theta )+(1-\beta_{i}) \end{array}\right..$$

Finally, the modified mass function is fused according to the synthetic rules of evidence theory.

## 5. Engineering application and discussion

### 5.1. Engineering background

In the western mountain area of China, the geological structure is complex and karst is widely distributed. A tunnel in this area is characterized by its large depth, long hole lines, high stress and high water pressure [69], which could easily cause water in-rush, or other geologically driven accidents, during construction.

**Figure 3.**
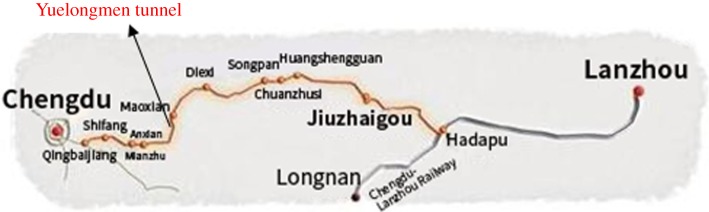
Chen-Lan railway and Yuelongmen tunnel.

The Yuelongmen tunnel of the Chengdu–Lanzhou railway (figure 3) is a control project for the railway, which has a length of approximately 20 km. It crosses multiple fault zones and the Peijiang River system, the region is seismically active and the geological environment is complex. Geological disasters such as water bursting, landslides, large deformation, rock bursts and other common geological disasters can occur. The method of simultaneous construction in multiple directions by cross-holes and inclined wells could accelerate the construction of the tunnel.

**Figure 4.**
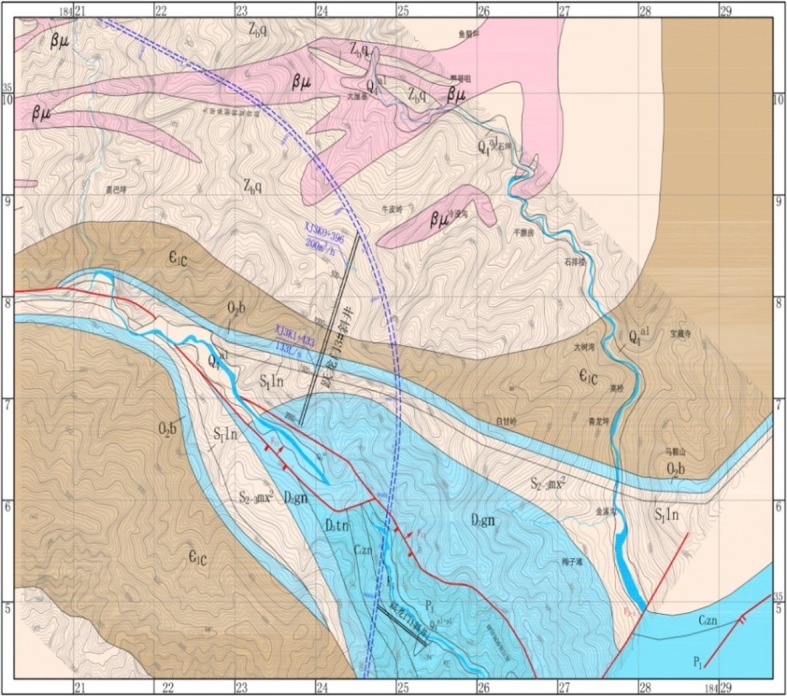
Regional azimuth map of the no. 3 inclined shaft in Yuelongmen tunnel.

The no. 3 inclined shaft of the Yuelongmen Tunnel is located on the left-hand side of the line. The starting point is XJ3K0, which is connected to the normal line at D2K97 + 700, and the terminus of the station is at XJ3K2 + 30. The length and maximum depth of the no. 3 inclined shaft are 2030 m and 872 m, respectively. The entrance to the inclined shaft is located on the left bank of Gaochuan River, and the entrance elevation is 1280 m (figure 4).

In addition, inclined shaft no. 3 is found to have the following characteristics:

1. The construction site is narrow and has inconvenient traffic volumes thereat.
2. Karst development includes broken rock and a high water content.
3. The shaft has an inclined well with an anti-slope construction that has unfavourable drainage tendencies.
4. The construction of the inclined shaft caused the nearby river to pour into the mountain.
5. There has been a change in the construction site of the no. 3 inclined shaft.

The above characteristics make the uncertain information more complex and the likelihood of geological disasters is higher, therefore, it is necessary to improve the quality of risk assessment and perfect risk control measures in the design stage. Taking the risk of water in-rush as an example, the no. 3 inclined shaft is evaluated and different measures are taken for different sections according to the assessment results.

### 5.2. Fuzzy risk analysis of tunnel water in-rush

The risk assessment of water in-rush of the no. 3 inclined well by a new fuzzy risk assessment method is presented here, and the water in-rush risk throughout the region can be better understood as a result. This can provide the basis for precise risk assessment during construction. The typical section from chainage XJ3K0 + 330 to XJ3K0 + 560 was selected to explain the proposed risk assessment model. The specific assessment process is described below, and all of the initial data used here are mainly based on the engineering geology and hydrogeological exploration report and a supplementary investigation report. Different interpretation of test results from different experts, and the results of the laboratory test are also used. On this basis, the data are extended to facilitate the construction of fuzzy numbers.

#### 5.2.1. The construction of fuzzy numbers for uncertain information

The karst development is the internal factor for controlling the formation of water-induced disasters. The concentration and distribution of karst water are basically consistent with the trend in karst development. The water-rich nature of the karst and the karst development morphology and scale depend on the lithology and hydrodynamic conditions of karst water occurrence and migration. The following essential factors (soluble rock *x*_1_, water pressure *x*_2_, groundwater circulation conditions *x*_3_ and surface water catchment conditions *x*_4_) are selected as risk assessment indices for water in-rush in such karst tunnels based on the aforementioned analysis and with reference to the relevant literature [3,70].

##### 5.2.1.1. Soluble rock

Soluble rock is the material foundation of karst development. Given the degree of difficulty of obtaining this type of information in the tunnel site, we determined the content of soluble rock mainly by considering the content of CaCO_3_ to evaluate rock solubility. The effect of rock structure on rock solubility was not considered in this work. The soluble rock samples were extracted in the field and related work was mainly analysed in the laboratory. A total of 30 samples were analysed, and the relevant results are shown in table 4.

**Table 4.** The test results of soluble rock content in the section of XJ3K0 + 330 ∼ XJ3K0 + 560 (%).

serial number	1	2	3	4	5	6	7	8	9	10
the test results	93.2	86.2	83.5	82.3	91.8	92	87.3	80.2	81.5	76.2
serial number	11	12	13	14	15	16	17	18	19	20
the test results	88.3	87.5	83.4	78.2	77.4	89.5	73.6	90.4	86.7	85.2
serial number	21	22	23	24	25	26	27	28	29	30
the test results	75.6	91.5	87	88.6	81.8	72.6	76.3	81.6	88.7	85.6

The data are normalized, then the average values (*m* = 84.12, *m*_1_ = 78.83 and *m*_2_ = 88.72) are found. The function of fuzzy number *x*_1_ is:

5.1 $$u(x_{i})=\left\{ \begin{array}{ll} (\frac{x_{i}-74.62}{8.50}{)}^{2}\;, & 74.62\leq x_{i}<83.12\\ 1, & 83.12\leq x_{i}<85.12\\ (\frac{92.31-x_{i}}{7.19}{)}^{2}\;, & 85.12\leq x_{i}<92.31\\ 0,\; & \mathrm{others} \end{array}\right..$$

The distribution of the other indicial (*x*_2_: water pressure at the tunnel face, *x*_3_: groundwater circulation conditions and *x*_4_: surface water catchment conditions) information can be obtained in the same way (see appendix D for details).

#### 5.2.2. Determination of the initial mass function

##### 5.2.2.1. Similarity measure

The similarity measure between the risk factors and the risk levels of the water in-rush can be obtained by using equation (3.4). Taking the rock solubility as an example, at first, the centre of gravity, perimeter and area of each risk assessment grade and risk evaluation index can be calculated as follows: Level A: *x*(*A*) = 9.58, *P*(*A*) = 46.21 and *A*(*A*) = 19; Level B: *x*(*B*) = 28.33, *P*(*B*) = 68.66 and *A*(*B*) = 22.67; Level C: *x*(*C*) = 48, *P*(*C*) = 77.05 and *A*(*C*) = 24.83; and Level D: *x*(*D*) = 82.05, *P*(*D*) = 97.93 and *A*(*D*) = 35.5; *x*_1_: *x*(*x*_1_) = 83.82, *l*(*x*_1_) = 37.34 and *A*(*x*_1_) = 7.23.

Then, the similarity measures are: *S*_11_ = *S*(*x*_1_, *A*) = 0.3276, *S*_12_ = *S*(*x*_1_, *B*) = 0.2834, *S*_13_ = *S*(*x*_1_, *C*) = 0.3096 and *S*_14_ = *S*(*x*_1_, *D*) = 0.3331).

Similarly, the others can also be obtained and the similarity measure matrix is as follows:

5.2 $$\left[\begin{array}{l} 0.3276\;\;0.2834\;\;0.3096\;\;0.3331\\ 0.3202\;\;0.5084\;\;0.6671\;\;0.7407\\ 0.3348\;\;0.5339\;\;0.6269\;\;0.6006\\ 0.3178\;\;0.2748\;\;0.3003\;\;0.3230 \end{array}\right].$$

##### 5.2.2.2. Mean, variance and induced function

For *x*_1_: *E*(*x*_1_) = 83.99, *D*(*x*_1_) = 2.75, *Q*(*x*_1_) = 37.33;

for *x*_2_: *E*(*x*_2_) = 71.01, *D*(*x*_2_) = 11.02, *Q*(*x*_2_) = 6.44;

for *x*_3_: *E*(*x*_3_) = 76.8, *D*(*x*_3_) = 8.19, *Q*(*x*_3_) = 9.38;

for *x*_4_: *E*(*x*_4_) = 73.9, *D*(*x*_4_) = 2.25 *Q*(*x*_3_) = 32.84.

##### 5.2.2.3. Initial mass function

Firstly, the ordered functions are as follows:

$$\mathrm{Order}(x_{1})=0.434,\mathrm{Order}(x_{2})=0.075,\mathrm{Order}(x_{3})=0.109,\mathrm{Order}(x_{4})=0.382.$$

The initial mass functions *m_ij_* are presented as follows:

$$\left\{ \begin{array}{l} m_{11}=m(x_{1},A)=0.2270,\;m_{12}=m(x_{1},B)=0.2532,\\ m_{13}=m(x_{1},C)=0.2829,\;m_{14}=m(x_{1},D)=0.2369\\ m_{21}=m(x_{2},A)=0.2496,\;m_{22}=m(x_{2},B)=0.2505,\\ m_{23}=m(x_{2},C)=0.2503,\;m_{24}=m(x_{2},D)=0.2496\\ m_{31}=m(x_{3},A)=0.2415,\;m_{32}=m(x_{3},B)=0.2530,\\ m_{33}=m(x_{3},C)=0.2542,\;m_{34}=m(x_{3},D)=0.2513\\ m_{41}=m(x_{4},A)=0.2467,\;m_{42}=m(x_{4},B)=0.2503,\\ m_{43}=m(x_{4},C)=0.2571,\;m_{44}=m(x_{4},D)=0.2458 \end{array}\right.$$

#### 5.2.3. Multi-source information fusion

Firstly, we determine the subjective and objective weights of each risk index using the aforementioned weight determination method.

##### 5.2.3.1. Subjective weights

According to the subjective weight method described herein, we invited several experts to provide an influence matrix with different indices, and the following matrix is obtained:

$$U=\left[\begin{array}{llll} \;(0.1,0.1,0.1,0.1) & \left(0.6,0.7,0.8,0.9\right) & \left(0.5,0.6,0.7,0.8\right) & \left(0.3,0.4,0.5,0.6\right)\\ \left(0.1,0.2,0.3,0.35\right) & \left(0.1,0.1,0.1,0.1\right) & \left(0.15,0.2,0.25,0.3\right) & \left(0.12,0.3,0.37,0.45\right)\\ \left(0.2,0.28,0.35,0.4\right) & \left(0.65,0.72,0.8,0.9\right) & \left(0.1,0.1,0.1,0.1\right) & \left(0.05,0.1,0.16,0.22\right)\\ \left(0.18,0.24,0.3,0.37\right) & \left(0.57,0.65,0.72,0.84\right) & \left(0.5,0.56,0.62,0.7\right) & \left(0.1,0.1,0.1,0.1\right) \end{array}\right].$$

According to equation (4.8), the fuzzy effect value of each index is:

$$\begin{array}{rl} & u_{1}=(0.2158,0.2871,0.3925,0.5092),\;u_{2}=(0.0676,0.1276,0.1906,0.1726)\\ & u_{3}=(0.1439,0.1914,0.2635,0.375),\;\;\;u_{4}=(0.1942,0.2472,0.3252,0.4467). \end{array}$$

The fuzzy expected value of each index can be determined by using equation (4.7):

*E*(*u*_1_) = 0.35115, *E*(*u*_2_) = 0.1396, *E*(*u*_3_) = 0.24345 and *E*(*u*_4_) = 0.3033.

Then the subjective weights are: $w_{u_{1}}^{*}=0.338$, $w_{u_{2}}^{*}=0.135$, $w_{u_{3}}^{*}=0.235$ and $w_{u_{4}}^{*}=0.292$.

##### 5.2.3.2. Objective weights

The correlation between different indices must first be determined.

$$R=\left[\begin{array}{cccc} 1 & 0.0498 & 0.9665 & -0.2054\\ 0.0498 & 1 & 0.3169 & -0.4908\\ 0.9665 & 0.3169 & 1 & -0.2911\\ -0.2054 & -0.4908 & -0.2911 & 1 \end{array}\right].$$

The objective weights can then be determined using equations (4.10) to (4.12):

$$w_{u_{1}}^{**}=0.2253,w_{u_{2}}^{**}=0.3210,w_{u_{3}}^{**}=0.1748\;\mathrm{and}\;w_{u_{4}}=0.2854$$

Finally the comprehensive weights are: $w_{u_{1}}=0.2817$, $w_{u_{2}}=0.2280$, $w_{u_{3}}=0.2049$ and $w_{u_{4}}=0.2854$.

##### 5.2.3.3. Information fusion

The maximum weight of *x*_4_ is the key factor, and the relative weights are determined to be $\beta_{x_{1}}=0.9870$, $\beta_{x_{2}}=0.7989$, $\beta_{x_{3}}=0.7179$ and $\beta_{x_{4}}=1$. The initial mass function can be modified using equation (4.14). The fusion results are summarized in table 5.

**Table 5.** Multi-source information fusion: water in-rush risk assessment.

weight coefficient *β*	*m*_1_ (A)	*m*_2_ (B)	*m*_3_ (C)	*m*_4_ (D)	*m* (*Θ*)
0.9870	0.2240	0.2499	0.2792	0.2338	0.0131
0.7989	0.1994	0.2001	0.2000	0.1994	0.2011
0.7179	0.1734	0.1816	0.1825	0.1804	0.2821
1	0.2467	0.2503	0.2571	0.2458	0
fusion results	0.2208	0.2521	0.2896	0.2375	0
evaluation result	C				

The fusion result in table 5 indicates that the risk level of water in-rush in the section of the tunnel running from XJ3K0 + 330 to XJ3K0 + 560 is C (high risk). The water in-rush risk in this area must be heeded during construction. The remaining sections of the no. 3 inclined shaft can be evaluated by the same analytical method mentioned (table 6).

**Table 6.** Evaluation of water in-rush risk in different sections of the no. 3 inclined shaft.

sections	geological description	risk assessment results of water in-rush
XJ3K0 + 000 ∼ XJ3K0 + 330	fracture development and groundwater circulation conditions are good	C
XJ3K0 + 330 ∼ XJ3K0 + 560	a karst fissure is developed, which is beneficial to the storage of groundwater	C
XJ3K0 + 560 ∼ XJ3K0 + 635	karst section: groundwater circulation conditions are good	B
XJ3K0 + 635 ∼ XJ3K0 + 815	the joint development is dense and the groundwater is dominated by fissure water in bedrock	B
XJ3K0 + 815 ∼ XJ3K1 + 090	the overall geological conditions are relatively stable	A
XJ3K1 + 090 ∼ XJ3K1 + 700	the karst is extremely developed and the groundwater storage conditions are good	C
XJ3K1 + 700 ∼ XJ3K2 + 030	karst is slightly developed, and the water influx is relatively small during monitoring	A

### 5.3. Discussion and verification of evaluation results

In table 6, there are three sections with the highest risk level (C). The length thereof is 1270 m, accounting for 62.7% of the total length, indicating that the overall water in-rush risk of the no. 3 inclined shaft is relatively high. According to the evaluation result, the decision-maker must make a corresponding plan against water in-rush risk and focus on high-risk sections of the tunnel. Owing to the constant updating of geological information during construction, the water in-rush risk may change, so special attention should be paid to changes in geological conditions during construction. The following will combine the actual situation of water burst risk in construction to illustrate the rationality of the proposed method.

On 3 March 2015, when the tunnel was excavated to XJ3K0 + 396, a small amount of water influx was noted. On 8 March 2015, the amount of water suddenly increased after a blasting operation: the construction team immediately stopped the operation, evacuated all non-essential personnel and used pumping equipment to reduce the tunnel water level. Experts were asked to assess the risk of water in-rush before further construction to improve the specified risk control scheme due to the high-risk evaluation at the design stage. One of the priority measures was to undertake advanced horizontal drilling in the tunnel face, which can not only add to the engineering geology and hydrogeological information, but also reduce the water pressure on the tunnel.

**Figure 5.**
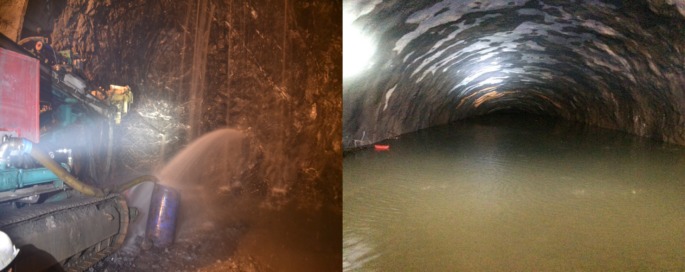
Water in-rush accident.

**Figure 6.**
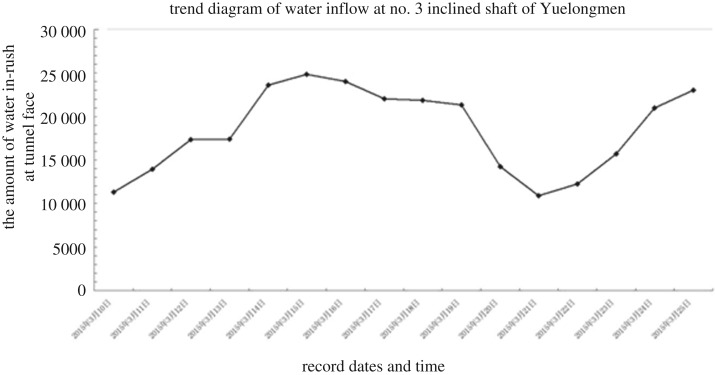
Water influx to the no. 3 inclined shaft.

On 10 March 2015, an advanced horizontal drill (total length 38 m) was applied to the face. When drilled to XJ3K0 + 382, there was a large amount of water found in the hole, and the water pressure increased significantly. When drilling to XJ3K0 + 356, the water pressure was too great to continue drilling. After withdrawing the drill rod, a water outburst occurred over about 15 m, and the water was clear. On 14 March 2015, another three holes were drilled, and the flow continued to increase, which resulted in the inundation of 70 m of the cave at a flow rate of about 1000 m^3^ h^−1^. At about 15.00 on 14 March 2015, there was no further increase in the amount of water in the cave. There were six sewage pumps active on the face, and the water flow was stable at about 800–900 m^3^ h^−1^ (figures 5 and 6).

At the design stage, the water in-rush risk from XJ3K0 + 330 to XJ3K0 + 560 is C (high risk), which is in line with the actual situation encountered during construction. It not only verifies the reliability of the proposed method, but also explains the rationality of the research thinking behind the safety risk assessment as applied to tunnel engineering, that is to emphasize the importance of risk assessment during construction, and pay attention to the role of the site investigation and design stages in the implementation of a safe risk control system.

## 6. Conclusion

With the implementation of ‘The Belt and Road’ strategy, more deeply buried, long tunnels will be constructed in complex geological conditions such as karst areas. Accurate risk analysis is an important guarantee for the safe construction of such tunnels. Based on the characteristics of tunnel engineering, it is necessary to implement an entire tunnel construction process risk analysis. Considering that current risk research is primarily focused on construction periods, there is no reliable risk assessment model for the design phase. To further improve tunnel engineering risk research and improve the risk control effect, this paper aims to construct a risk assessment model which fully reflects the risk characteristics of the design stage. The main conclusions are as follows:

(1) Based on the characteristics of information deficiency in the design stage and its role in the risk analysis system by providing basic information guidance for risk analysis during construction, it is reasonable and necessary to construct a semi-quantitative risk assessment model. (2) The fuzzy risk analysis model based on the similarity measure, which has been a commonly used semi-quantitative evaluation method, has some deficiencies including information loss and inability to adapt to nonlinear characteristics. To improve the deficiencies of the existing models, an improved fuzzy risk assessment model based on similarity measures and evidence theory has been proposed. (3) The proposed model was used to assess water in-rush risk in the design stage of the no. 3 inclined shaft of the Yuelongmen tunnel. The tunnel is divided into different sections according to the water in-rush risk levels, and the high-risk area of water in-rush can be focused on during construction. Effective risk control measures or schemes are amended or proposed based on the accurate analysis method, such as probability risk assessment during construction. Through semi-quantitative risk analysis in the design stage, the efficiency and effectiveness of risk control during construction can be improved.

(4) The constructed fuzzy risk assessment model is an important part of a risk analysis system of the whole construction process, which could deal with different types of risk information in the design stage of tunnel engineering, such as qualitative language, fuzzy information and interval information. The model is advantageous compared to traditional techniques using statistical data and the approach can be seen as a useful complement to existing risk analysis methods.

## Data accessibility

This theoretical paper does not rely on empirical data. All the plots can be generated following the equations and instructions provided in the paper.

## Appendix A

The arithmetic operations between the generalized trapezoidal fuzzy numbers *A* and *B* are as follows

A 1 $$\begin{array}{rl} \tilde{A}⊗\tilde{B} & =(a_{1},b_{1},c_{1},d_{1};w_{1})⊗(a_{2},b_{2},c_{2},d_{2};w_{2})\\ & \approx (a_{1}a_{2},b_{1}b_{2},c_{1}c_{2},d_{1}d_{2};\min(w_{1},w_{2})), \end{array}$$

A 2 $$\begin{array}{rl} \tilde{A}⊕\tilde{B} & =(a_{1},b_{1},c_{1},d_{1};w_{1})⊗(a_{2},b_{2},c_{2},d_{2};w_{2})\\ & =(a_{1}+a_{2}-a_{1}a_{2},b_{1}+b_{2}-b_{1}b_{2},c_{1}+c_{2}-c_{1}c_{2},d_{1}+d_{2}-d_{1}d_{2};\min(w_{1},w_{2})) \end{array}$$

and

A 3 $$\begin{array}{rl} \tilde{A}\text{Ø}\tilde{B} & =(a_{1},b_{1},c_{1},d_{1};w_{1})\;\text{Ø}\;(a_{2},b_{2},c_{2},d_{2};w_{2})\\ & \approx (\frac{a_{1}}{d_{2}},\frac{b_{1}}{c_{2}},\frac{c_{1}}{b_{2}},\frac{d_{1}}{a_{2}};\;\min(w_{1},w_{2})), \end{array}$$

where

$$\frac{a}{b}=\{\begin{array}{ll} \frac{a}{b}, & \mathrm{if}\;a<b\\ 1, & \mathrm{otherwise} \end{array}$$

A 4 $$\begin{array}{rl} \tilde{A}\Theta \tilde{B} & =(a_{1},b_{1},c_{1},d_{1};w_{1})⊗(a_{2},b_{2},c_{2},d_{2};w_{2})\\ & =(a_{1}-d_{2},b_{1}-c_{2},c_{1}-b_{2},d_{1}-a_{2};\;\min(w_{1},w_{2})). \end{array}$$

## Appendix B

Based on a previous study [60], Chen & Chen [61] proposed a new method for calculating the similarity measure between fuzzy numbers using the concept of centre of gravity theory (COG).

B 1 $$S(\tilde{A},\tilde{B})=\left(1-\frac{\sum_{i=1}^{4}\left|a_{i}-b_{i}\right|}{4}\right)\times {\left(1-|x_{\tilde{A}}^{*}-x_{\tilde{B}}^{*}|\right)}^{\;f\;(l_{\tilde{A}},l_{\tilde{B}})}\times \frac{\min(y_{\tilde{A}}^{*},y_{\tilde{B}}^{*})}{\max(y_{\tilde{A}}^{*},y_{\tilde{B}}^{*})},$$

where

$$f\;(l_{\tilde{A}},l_{\tilde{B}})=\{\begin{array}{ll} 1 & \mathrm{if}\;\;0<\frac{l_{\tilde{A}}+l_{\tilde{B}}}{2}\leq 1\\ 0 & \mathrm{if}\;\;\frac{l_{\tilde{A}}+l_{\tilde{B}}}{2}=0 \end{array},\;l_{\tilde{A}}=a_{4}-a_{1},\;l_{\tilde{B}}=b_{4}-b_{1}.$$

In 2009, based on the characteristics of geometric distance, perimeter and height of fuzzy numbers, Wei & Chen [62] proposed a new method to calculate the similarity measure.

B 2 $$S(\tilde{A},\tilde{B})=\left(1-\frac{\sum_{i=1}^{4}\left|a_{i}-b_{i}\right|}{4}\right)\times \frac{\min(P(\tilde{A}),P(\tilde{B}))+\min(w_{1},w_{2})}{\max(P(\tilde{A}),P(\tilde{B}))+\max(w_{1},w_{2})},$$

where $P(\tilde{A})$ and $P(\tilde{B})$ are the perimeters of the fuzzy numbers *Ã* and *B̃*, and the formulae are written as follows:

B 3 $$P(\tilde{A})=\sqrt{{\left(a_{1}-a_{2}\right)}^{2}+w_{1}^{2}}+\sqrt{{\left(a_{3}-a_{4}\right)}^{2}+w_{1}^{2}}+(a_{3}-a_{2})+(a_{4}-a_{1})$$

and

B 4 $$P(\tilde{A})=\sqrt{{\left(b_{1}-b_{2}\right)}^{2}+w_{1}^{2}}+\sqrt{{\left(b_{3}-b_{4}\right)}^{2}+w_{1}^{2}}+(b_{3}-b_{2})+(b_{4}-b_{1}).$$

In 2010, Xu *et al*. [63] presented a new similarity measure for fuzzy numbers based on distance and COG.

B 5 $$S(\tilde{A},\tilde{B})=1-\frac{1}{2}\times \left|\frac{\sum_{i=1}^{4}\left|a_{i}-b_{i}\right|}{4}+d^{\prime }(\tilde{A},\tilde{B})\right|,$$

where $d^{\prime }(\tilde{A},\tilde{B})$ is the distance of the COG points of fuzzy numbers, as follows:

$d^{\prime }(\tilde{A},\tilde{B})=\sqrt{{\left(x_{\tilde{A}}^{*}-x_{\tilde{B}}^{*}\right)}^{2}+{\left(y_{\tilde{A}}^{*}-y_{\tilde{B}}^{*}\right)}^{2}}/\sqrt{1.25}$. $\left(x_{\tilde{A}}^{*},y_{\tilde{B}}^{*}\right)$ are the coordinates of COG, and the expressions are expressed as follows.

B 6 $$x_{\tilde{A}}^{*}=\{\begin{array}{ll} \frac{y_{\tilde{A}}^{*}\times (a_{3}+a_{2})+(a_{4}+a_{1})\times (w_{\tilde{A}}-y_{\tilde{A}}^{*})}{2w_{\tilde{A}}}, & \mathrm{if}\;w_{\tilde{A}}\neq 0\\ \frac{a_{4}+a}{2}, & \mathrm{if}\;w_{\tilde{A}}=0 \end{array}$$

B 7 $$y_{\tilde{A}}^{*}=\{\begin{array}{ll} \frac{w_{\tilde{A}}\times ((a_{3}-a_{2}/a_{4}-a_{1})+2)}{6}, & \mathrm{if}\;\;a_{4}\neq a_{1}\;\\ \frac{w_{\tilde{A}}}{2},\; & \mathrm{if}\;\;a_{4}=a \end{array}$$

Hejazi *et al*. [64] proposed a novel method of fuzzy risk analysis based on a new similarity measure of generalized fuzzy numbers, which considers many of their features, such as area, perimeter, height and geometric distance and could potentially overcome the limitations of the other methods. The formula of similarity measure is as follows.

B 8 $$S(\tilde{A},\tilde{B})=\left(1-\frac{\sum_{i=1}^{4}\left|a_{i}-b_{i}\right|}{4}\right)\times \frac{\min(P(\tilde{A}),P(\tilde{B}))}{\max(P(\tilde{A}),P(\tilde{B}))}\times \frac{\min(A(\tilde{A}),A(\tilde{B}))+\min(w_{\tilde{A}},w_{\tilde{B}})}{\max(A(\tilde{A}),A(\tilde{B}))+\max(w_{\tilde{A}},w_{\tilde{B}})},$$

where $A(\tilde{A})$ and $A(\tilde{B})$ are the areas of *Ã* and *B̃*, respectively, and the formulae are as follows:

$$A(\tilde{A})=\frac{w_{\tilde{A}}\times (a_{3}-a_{2}+a_{4}-a_{1})}{2},\;A(\tilde{B})=\frac{w_{\tilde{B}}\times (b_{3}-b_{2}+b_{4}-b_{1})}{2}.$$

## Appendix C

See table 7.

**Table 7.** Scale method and its definition.

impact scale	*m_ij_*
0.1	index *u_i_* does not influence *u_j_*
0.3	index *u_i_* produces a slight effect on *u_j_*
0.5	index *u_i_* produces a general effect on *u_j_*
0.7	index *u_i_* produces a great effect on *u_j_*
0.9	index *u_i_* produces a significant effect on *u_j_*
0.2, 0.4	index *u_i_* influences *u_j_*
0.6, 0.8	the degree is in the middle of the results for 0.5–0.9

## Appendix D

The distributions of the other indexes (*x*_2_: water pressure at the tunnel face, *x*_3_: groundwater circulation conditions, *x*_4_: surface water catchment conditions) information are as follows.

(1) Water pressure at the tunnel face (*x*_2_)

Water pressure is an important factor for water in-rush. Table 8 presents the data obtained on site according to the regular measurement of the drilling water pressure.

**Table 8.** Monitoring data of the water pressure (MPa).

58	70.75	83.5	64	91.25	55.75	84.25	75.5	68.75	56
70.25	60.75	73.75	94	95.5	66.25	55.5	60.75	68.25	66.75
59.5	65	67	53.25	48.75	71.75	82	86.5	67.25	46.5

The function of fuzzy number of *x*_2_ is:

D 1 $$\pi (x_{i})=\{\begin{array}{ll} {\left(\frac{x_{i}-52.98}{14.92}\right)}^{2}, & 52.98\leq x_{i}<67.9\\ 1, & 67.9\leq x_{i}<69.9\\ {\left(\frac{93.26-x_{i}}{23.36}\right)}^{2}, & 69.9\leq x_{i}<93.26\\ 0. & \mathrm{others} \end{array}.$$

(2) Groundwater circulation conditions (*x*_3_).

The erodibility of water flow is one of the basic conditions for karst development. The dissolution occurs first in the karst and water interface. The soluble rock dissolution reaches a saturation state if the dissolved ions cannot move quickly, thereby preventing further dissolution. Table 9 shows the monitoring data of the groundwater circulation condition.

**Table 9.** Monitoring data of the groundwater circulation condition.

−0.12	−0.18	−0.02	0.03	0.25	0.02	0.3	0.72	0.08	−0.29
−0.07	−0.14	0.21	0.33	−0.05	0.1	0.36	−0.22	−0.15	−0.06
0.18	0.43	0.12	0.17	0.24	0.62	−0.05	−0.09	0.19	0.69

The function of the fuzzy number of *x*_3_ is:

D 2 $$\pi (x_{i})=\{\begin{array}{ll} {\left(\frac{x_{i}-57.84}{18.44}\right)}^{2}, & 57.84\leq x_{i}<76.28\\ 1, & 76.28\leq x_{i}<78.28\\ {\left(\frac{91.92-x_{i}}{13.64}\right)}^{2}, & 78.28\leq x_{i}\leq 91.92\\ 0, & \mathrm{others} \end{array}.$$

(3) Surface water catchment conditions (*x*_4_)

The karst landform conditions and the formation and development of underground karst spaces promote and interact with each other. The percentage of the negative terrain area (*x*_41_) and the slope gradient (*x*_42_) were selected to evaluate the quality of the surface water catchment conditions. Table 10 presents the monitoring data of the surface water catchment condition.

**Table 10.** Monitoring data of the surface water catchment condition.

percentage of the negative terrain area %	65.3	63.2	69.3	71.5	75.2	68.4	67.7	64	73.2	73.8
	62.5	66.9	74.7	78.3	76.2	69.7	70.9	74.3	72.7	67.4
	58.5	63.4	65.2	73.8	69.8	77.2	73.8	75.3	80.4	62.5
slope gradient (°)	15.7	16.8	17.7	19.4	18.5	20.3	23.5	21.4	25.7	14.7
	23.4	24.6	26.3	13.4	22.8	21.2	18.7	27.2	15.2	13.3
	12.6	14.6	16.3	18.4	15.2	17.3	11.4	13.7	17.7	13.8

The functions of the fuzzy number of *x*_41_ and *x*_42_ are:

D 3 $$\pi (x_{i})=\{\begin{array}{ll} {\left(\frac{x_{i}-62.01}{7.16}\right)}^{2}, & 62.01\leq x_{i}<69.17\\ 1, & 69.17\leq x_{i}<71.17\\ {\left(\frac{78.33-x_{i}}{7.16}\right)}^{2}, & 71.17\leq x_{i}<78.33\\ 0, & \mathrm{others} \end{array}$$

and

D 4 $$\pi (x_{i})=\{\begin{array}{ll} {\left(\frac{x_{i}-71.93}{6.7}\right)}^{2}, & 71.93\leq x_{i}<78.63\\ 1, & 78.63\leq x_{i}<80.63\\ {\left(\frac{86.11-x_{i}}{5.48}\right)}^{2}, & 80.63\leq x_{i}<86.11\\ 0 & \mathrm{others} \end{array}.$$

The distribution of the surface water catchment conditions *x*_4_ can be synthesized with *x*_41_ and *x*_42_ by linear weights.
